# Temperature as a Metabolic Signal Linking Neural and Endocrine Circuits to Energy Homeostasis

**DOI:** 10.3390/ijms27093765

**Published:** 2026-04-23

**Authors:** Xueying Mo, Young-Bum Kim, Cheng Huang, Shengjie Fan

**Affiliations:** 1 School of Pharmacy, Shanghai University of Traditional Chinese Medicine, Shanghai 201203, China; carolinemok@163.com; 2 Division of Endocrinology, Diabetes, Metabolism, Beth Israel Deaconess Medical Center, Harvard Medical School, Boston, MA 02115, USA; ykim2@bidmc.harvard.edu

**Keywords:** thermosensation, appetite regulation, body weight regulation, metabolic regulation, neuroendocrine control

## Abstract

Ambient temperature is a continuous environmental input that affects energy homeostasis through integrated physiological programs. In mammals, thermal cues detected by cutaneous and visceral sensors are conveyed through spinal, vagal, and sympathetic pathways. They are complemented by circulating mediators from the gut, liver, and adipose tissue. These signals converge on brainstem–hypothalamic networks, including the preoptic area and arcuate nucleus, to coordinate feeding behavior, thermogenesis, vasomotor tone, and endocrine outputs. Recent circuit-mapping and single-cell approaches have refined the cellular logic governing the distinct architectures of cold- and heat-defense programs. This review synthesizes these advances to illustrate how a plastic effector network maintains systemic energy homeostasis. Finally, we highlight the translational implications of these thermosensory mechanisms for treating obesity and type 2 diabetes.

## 1. Introduction

Ambient temperature is a pervasive environmental variable that shapes the bioenergetic strategies of endotherms. To defend core temperature, mammals engage coordinated physiological and behavioral responses that couple thermoregulation to energy intake and expenditure [1,2,3]. Central to this regulation is the “thermoneutral zone (TNZ)”—a range where basal metabolic rate is minimal. While evolutionarily efficient, modern built environments often “clamp” humans in prolonged thermoneutrality, potentially inducing “metabolic inertia” and blunting adaptive capacity [4].

Deviations from TNZ trigger distinct survival strategies: the cold-defense and heat-defense programs. The cold-defense program is energetically expensive, driving sympathetic activation for vasoconstriction and thermogenesis. Conversely, the heat-defense program prioritizes heat dissipation and metabolic suppression to prevent hyperthermia [5,6]. These opposing programs are governed by a hierarchical network where peripheral cues are conveyed to key integrators: Thermal information from cutaneous and visceral afferents is conveyed via spinal and vagal pathways to brainstem and hypothalamic circuits [7,8]. Although previous studies have systematically elucidated the neural mechanisms underlying long-term temperature-adaptive changes in mammals in response to cold and metabolic demands [9]. Within this network, key nodes—including the preoptic area (POA) and arcuate nucleus (ARC)—integrate thermal inputs with nutrient and hormonal inputs to coordinate feeding, vasomotor tone, and systemic metabolism [10].

Despite the robust impact of ambient temperature on metabolism in experimental models, translational strategies that leverage thermal biology for obesity and type 2 diabetes remain in early stages. This review highlights recent advances in circuit-mapping and single-cell profiling to outline the peripheral sensors, neuroendocrine relays, and central integrator circuits linking temperature to energy homeostasis. We also discuss mechanisms of sensing, integration, and effector execution and highlight key challenges for translation, including species differences and the confounding impact of thermoneutrality.

## 2. Peripheral Temperature Sensing: Key Organs and Molecular Signaling Pathways

**Figure 1:**
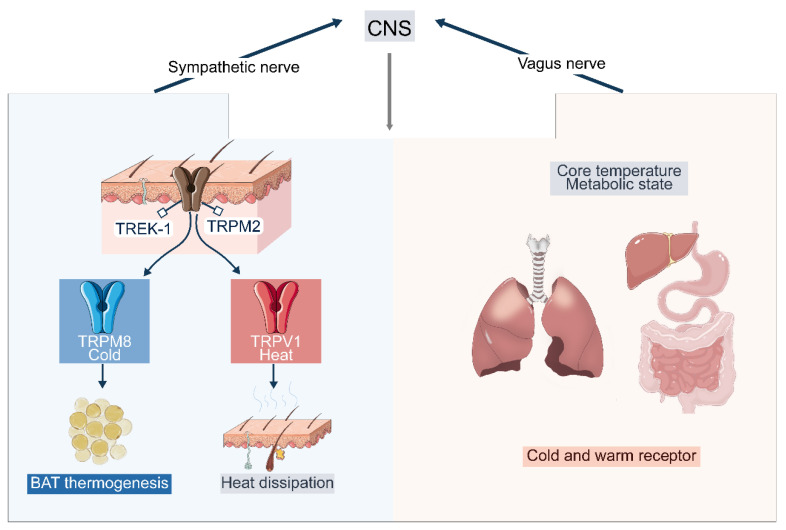
Schematic of peripheral temperature sensing. This figure illustrates the mechanisms of thermal perception in the skin and visceral organs. Cold- and heat-sensitive receptors (e.g., TRPM8, TRPV1 and TRPM2) are distributed in the epidermis and in visceral tissues such as the lung, liver, stomach, and intestine, where they respond to changes in ambient and core temperature. These peripheral sensors convey thermal information through neural afferent pathways to initiate physiological responses, including brown adipose tissue (BAT) thermogenesis and heat dissipation, thereby contributing to the regulation of body temperature and energy homeostasis. Arrows indicate the direction of information flow. This figure was created using Adobe Illustrator (version 2020) and Procreate (version 4.4.10).

Energy homeostasis begins with the detection of ambient and core temperature by specialized sensors. In mammals, these cues are captured by a diverse array of cutaneous and visceral receptors that initiate distinct physiological programs (Figure 1).

### 2.1. Skin: The “Frontline” of the Body’s Temperature Perception

Cutaneous thermosensation serves as the body’s primary interface with the environment and acts as a metabolic gatekeeper, converting thermal energy into neural codes. At the molecular level, this process is governed by the transient receptor potential (TRP) channel family, which functions as a core sensory unit for both heat and cold [11]. TRPM8 channels are well-established as the primary sensors for cold-defense, triggering nonshivering thermogenesis in BAT [12,13,14,15], while TRPV1 and other heat-sensitive channels signal for cooling responses and appetite suppression [16,17]. These signals travel from primary sensory neurons in the dorsal root ganglia to the spinal cord, eventually reaching the POA of the hypothalamus to coordinate systemic energy balance [7,18,19].

Despite the clear roles of specific channels such as TRPM8, significant uncertainties remain about how the central nervous system distinguishes between localized and systemic thermal cues. While TRP channels define fundamental activation thresholds, it is not yet fully understood how these peripheral inputs are weighted against an animal’s internal nutrient status to drive complex behaviors. Furthermore, the role of auxiliary pathways, such as two-pore-domain potassium channels like TREK-1 [20,21], in fine-tuning these thermal sensitivities remains a subject of active debate. This highlights a conceptual gap in our understanding of how the brain integrates multiple, sometimes conflicting, and sensory inputs to maintain a stable metabolic state.

These uncertainties point toward several critical testable gaps that future research should address to improve translational outcomes. Specifically, it remains unclear how a single cold signal can simultaneously drive opposing metabolic outcomes, such as increasing food intake while also accelerating energy expenditure through BAT thermogenesis. In addition, the impact of chronic thermal exposure on the recalibration of these sensory thresholds in the context of obesity and type 2 diabetes is largely unknown. Bridging these gaps, particularly by validating whether these rodent-derived circuit models hold in humans under various thermal conditions, is essential for developing temperature-based therapeutic interventions.

### 2.2. Visceral Temperature Sensing: Core Thermal Signals and Metabolic Integration

In contrast to cutaneous sensors, visceral organs such as the gastrointestinal tract, liver, and respiratory system mainly signal core temperature and metabolic state via vagal afferents, with both warm- and cold-sensitive receptors identified in these regions [22,23,24,25]. Human studies further demonstrate that ingestion of fluids at different temperatures can modulate thermoregulatory responses even without changes in skin or core temperature [26], providing functional evidence for visceral thermosensation.

Despite these findings, major gaps remain in our understanding of visceral thermosensation and its role in metabolic regulation. For example, in mice, cold exposure suppresses *ob* gene expression in white adipose tissue through sympathetic signaling [27], and PACAP receptors in sympathetic ganglia exhibit temperature-sensitive expression [28], suggesting that thermal information can influence metabolic control through multiple pathways and hierarchical levels. Nevertheless, it is still unknown how visceral thermal inputs control metabolism in various physiological states, whether skin and visceral temperature sensing function in parallel or in a directed manner, and whether thermal signals are transferred from the skin to internal organs to influence metabolism.

## 3. Central Nervous System Integration and Command Execution

**Figure 2:**
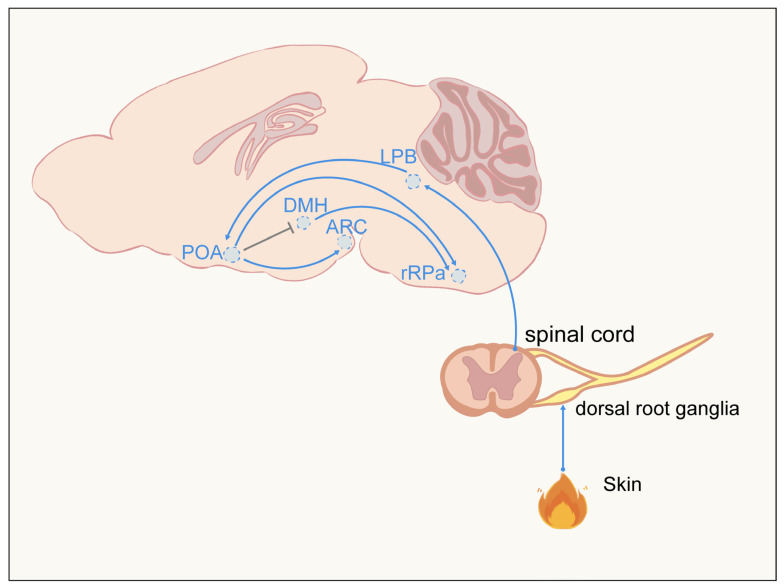
Afferent and efferent pathways of temperature signaling. Temperature signals are detected by primary sensory ganglia in the skin and subsequently relayed to the dorsal horn of the spinal cord, the lateral parabrachial nucleus (LPB), and the POA. Thermoregulatory neurons in the POA project to the DMH and the rRPa, with the rRPa also receiving strong inputs from the DMH. Blue, efferent pathways; gray, bidirectional communication. This figure was created using Adobe Illustrator (version 2020) and Procreate (version 4.4.10).

Energy homeostasis depends on the integration of thermal information and the generation of regulatory commands by the central nervous system. Diverse peripheral thermosensors first detect signals of ambient and core temperature and transmit them to the brain, where these inputs are converged, compared, and integrated and subsequently transformed into coordinated autonomic, endocrine, and behavioral outputs that collectively maintain systemic energy balance (Figure 2).

### 3.1. POA: Central Hub for Thermal Integration and Comparison

Since the seminal observation by Magoun et al. [29] that warming the POA induces hypothermia, extensive lesion and pharmacological studies have demonstrated that impairment of POA function disrupts autonomic thermoregulatory responses to both heat and cold exposure, while largely preserving behavioral thermoregulation such as warmth-seeking and cold avoidance [30,31,32,33]. These findings establish the POA as a central node in autonomic thermoregulation. POA neurons are also capable of integrating peripheral thermal inputs with central signals reflecting local brain temperature changes during locomotion or fever [34], thereby acting as a functional “comparator” that translates temperature deviations into continuously adjustable physiological commands [35,36]. However, despite the well-recognized role of the POA as a thermoregulatory center, the fundamental activation thresholds of POA neurons and the mechanisms by which they discriminate between peripheral and core temperature signals to generate appropriate and coordinated physiological outputs remain poorly defined.

### 3.2. PBN: Sensory Relay Hub

Thermal signals detected in the skin are transmitted by primary sensory neurons to the dorsal horn of the spinal cord, where neurons of the lateral parabrachial nucleus integrate excitatory inputs from the spinal cord and relay temperature information to the POA [18,37]. Along this pathway, the PBN and associated brainstem nuclei not only transmit thermal information but also distribute it to hypothalamic and limbic structures involved in arousal, emotion, and autonomic regulation. For example, estrogen and NPVF signaling in the medial amygdala modulate physical activity, thermogenesis, and glucose metabolism in a temperature-dependent manner [38,39]. Activated PBN neurons can optimize core body temperature by promoting vasodilation and reducing physical activity [40,41] and food intake [40].

### 3.3. ARC: Metabolic Integration and Output Node

The ARC functions as a metabolic integration and output node that directly links thermoregulation to energy balance after processing in the POA and PBN. The ARC is a highly energy-sensitive hypothalamic region containing pro-opiomelanocortin (POMC) neurons and agouti-related peptide (AgRP)/neuropeptide Y (NPY) neurons. These neuronal populations integrate peripheral metabolic and thermal signals to finely regulate feeding behavior and thermogenesis [42,43]. Thermosensitive neurons in the medial preoptic area project to AgRP-expressing orexigenic neurons in the ARC during cold exposure, causing cold-induced feeding; the medial preoptic area-ARC pathway’s activation increases feeding responses while its inhibition reduces cold-evoked hyperphagia [10]. In recent years, single-cell transcriptomic analyses have identified a distinct population of GABAergic neurons within the ARC-Crabp1-expressing neurons that do not express POMC or AgRP.

### 3.4. Downstream Effectors and Peripheral Actions

Temperature information integrated along the POA–PBN–ARC axis is relayed through intermediary nodes such as the dorsomedial hypothalamus (DMH) and ultimately engages key output structures, including the rostral raphe pallidus (rRPa), to regulate autonomic responses. The rRPa is a principal downstream hub controlling BAT thermogenesis, with its activation inducing heat production and its inhibition abolishing this process [44,45,46]. The rRPa is densely innervated by the DMH, which in turn receives direct input from the POA [47,48,49]. These effector pathways act on peripheral organs to elicit autonomic responses, such as suppressing thermogenesis and promoting heat dissipation through sweating and vasodilation [50,51,52]. This circuitry reshapes the organism’s energy expenditure state and preserves thermal homeostasis by coordinating heat production and loss.

In summary, the POA–PBN–ARC axis represents a core central integrative pathway linking sensory thermal inputs to metabolic decision-making. The POA functions as a physiological “comparator” that integrates peripheral and central temperature signals to generate regulatory commands; the PBN and associated brainstem nuclei relay thermal information and distribute it to autonomic and emotion-related circuits; and the ARC directly couples temperature signals to energy-homeostatic control of feeding and thermogenesis. In recent years, single-cell transcriptomic analyses have further resolved the molecular heterogeneity of these central nuclei. In the POA, a population of thermosensitive neurons characterized by TRPC4 expression has been identified, suggesting that TRPC4 functions as a central temperature-sensing component involved in thermal signal encoding [35]. In the ARC, a distinct group of Crabp1-expressing GABAergic neurons has been discovered that does not belong to the classical POMC or AgRP lineages; nevertheless, these neurons exert profound effects on systemic energy metabolism by regulating body temperature, locomotor activity, and BAT thermogenesis [53]. Recent advances in single-cell transcriptomic analysis have enabled the identification of previously unrecognized neuronal subpopulations in the hypothalamus at unprecedented molecular resolution and have also facilitated the construction of detailed cellular atlases [54,55,56]. Collectively, these advances are reshaping our understanding of the functional organization of neural circuits that regulate metabolism, particularly at increasingly refined cellular resolution.

## 4. Effector: Autonomic, Endocrine, and Behavioral Control of Thermometabolic Responses

After central integration, thermal information is transmitted to a plastic effector network that systemically regulates thermogenesis (BAT), vasomotor tone, feeding behavior, and endocrine function. The coordinated actions of these components constitute the executive basis for the integrated control of metabolism and body temperature.

### 4.1. BAT Thermogenesis: A Key Effector of Heat Production

One of the most straightforward and thoroughly researched effector mechanisms in reactions to temperature changes is BAT thermogenesis. BAT converts chemical energy directly into heat through uncoupling protein 1 (UCP1)-mediated mitochondrial uncoupling, a process that primarily depends on fatty acids mobilized by sympathetic stimulation [57,58]. BAT exhibits marked cellular heterogeneity: thermogenically active subpopulations with high UCP1 expression (BA-Hs) are preferentially activated by cold exposure, whereas subpopulations with low UCP1 expression (BA-Ls) can be converted into BA-Hs under cold conditions [59]. Beyond direct heat production, BAT also secretes peptides, lipids, and extracellular vesicles that regulate adipogenesis, glucose and lipid metabolism, inflammation, and thermogenesis, thereby influencing appetite, insulin sensitivity, and systemic energy homeostasis [60]. For example, leptin expression in interscapular BAT (iBAT) displays circadian rhythmicity and fluctuates in response to nutritional and thermal cues. Interscapular BAT-derived leptin activates leptin signaling within sympathetic ganglia via paracrine mechanisms and subsequently modulates hypothalamic POMC neuron activity, coupling energy intake to temperature signals [61,62].

### 4.2. Vasomotor Tone: A Dynamic Effector of Heat Conservation and Dissipation

The skin is an effective thermoregulatory organ because of its dense superficial venous plexuses, which allow for precise control of body temperature through dynamic regulation of blood flow. This function is mainly controlled by sympathetic vasomotor nerves and is expressed through two reflex mechanisms: (a) reflex cutaneous vasodilation, whereby an increase in body temperature triggers sympathetic pathways that cause cutaneous blood vessels to dilate, increasing peripheral blood flow and facilitating heat dissipation to the environment; and (b) reflex cutaneous vasoconstriction, where a decrease in skin or core temperature triggers noradrenergic sympathetic fibers, causing cutaneous vessel constriction and decreased blood flow to conserve heat [3]. Warm stimulation triggers thermosensitive neurons in the POA, which suppress sympathetic output from the DMH and the rRPa, causing cutaneous vasodilation and improving heat loss [47,50,51]. Accordingly, regulation of vasomotor tone represents a key physiological interface linking temperature sensing, autonomic output, and energy homeostasis.

### 4.3. Feeding: A Temperature-Responsive Energy Effector

When ambient temperature falls below the TNZ, homeothermic mammals must expend additional energy to maintain body temperature homeostasis. To meet this increasing energetic demand, animals typically enhance foraging and feeding behavior; the colder the environment, the greater the food intake required to sustain core body temperature [63,64,65]. For example, laboratory mice housed at 4 °C can nearly double their daily food intake, with thermogenic expenditure accounting for approximately 60% of total energy consumption [66]. The feeding response is highly context-dependent, and exposure to cold does not always result in increased feeding. In seasonal species adapted to hibernation, appetite exhibits cyclical “feast–fast” patterns, initiated only when sufficient fat stores are available [67,68]. For instance, in Daurian ground squirrels, late-summer cold exposure elevates hypothalamic orexigenic neuropeptides and UCP1 expression in BAT, yet does not increase food intake, indicating that this process is strongly regulated by annual rhythmicity [69]. In contrast, under extreme heat conditions, reduced thermogenic demand combined with increased heat-dissipation burden preferentially suppresses food intake, an effect that can override feeding state per se [70,71]. A population-based study in China further showed that for each 1 °C increase in ambient temperature, average food intake decreases by 0.11% [72]. Temperature also shapes food preference, with individuals favoring hot foods in cold environments and cold foods during heat exposure [73].

Unlike thermogenesis and vasomotor control, which are predominantly governed by autonomic mechanisms, feeding regulation relies more heavily on hypothalamic metabolic nuclei, particularly the ARC. Within the ARC, AgRP/NPY and POMC neuronal populations play central roles in temperature-dependent modulation of food intake [42,43,74]. Interestingly, thermal signals can also directly control feeding through a POA-ARC pathway; even in the absence of a significant increase in energetic demand, selective activation of this circuit is sufficient to encourage feeding [10]. These findings indicate that temperature signals influence feeding not only indirectly through changes in energy expenditure but also by directly engaging canonical hypothalamic feeding circuits.

Beyond neural pathways, temperature-dependent regulation of feeding is further shaped by peripheral metabolic and endocrine signals. Changes in ambient temperature can alter the circulating levels or central sensitivity of hormones such as leptin [75], thyroid hormones [76,77], and glucocorticoids [78,79,80,81,82]. Consequently, feeding behavior should not be viewed as a simple reflexive response to temperature, but rather as an integrated output arising from central thermal processing, metabolic state, and endocrine feedback.

### 4.4. Endocrine Axes: Temperature-Responsive Hormonal Effectors

Temperature changes not only regulate feeding directly via neural pathways but also serve as stress signals that activate endocrine axes, thereby indirectly influencing energy homeostasis. The hypothalamic–pituitary–adrenal (HPA) axis plays a central role in this process: thermal stress triggers hypothalamic neuronal activity, promoting the release of corticotropin-releasing hormone (CRH) and consequently elevating glucocorticoid levels (cortisol in humans, corticosterone in rodents) [78,79,80]. Both acute heat exposure and cold stress can increase circulating adrenocorticotropic hormone and glucocorticoid concentrations in rats [81,82]. Moreover, sustained elevation of glucocorticoids, particularly cortisol, is well known to promote central fat accumulation, induce insulin resistance, and enhance lipid deposition in visceral adipose depots, all of which constitute key features of metabolic syndrome (Mets) and obesity [83,84]. Glucocorticoids also regulate feeding behavior and food reward by modulating leptin, insulin, and NPY signaling. In the short term, insulin and leptin counteract glucocorticoid effects to maintain energy balance; however, under sustained thermal stress, this balance can be disrupted, leading to increased appetite, enhanced energy storage, and visceral fat accumulation [85].

At the same time, acute thermal stress activates the sympatho-adrenal system. In both humans and rodents, acute stress elevates epinephrine secretion, and sympathetic activation induces transient hyperglycemia, supporting energy demands during stress through increased glucose uptake in muscles and elevated heart and respiratory rates [86]. Acute cold exposure also significantly increases norepinephrine turnover in interscapular BAT [87], further enhancing thermogenesis and energy mobilization.

Thyroid hormones (TH) are another key endocrine signal regulating energy metabolism during thermal stress. Cold and other physical stressors activate the hypothalamic-pituitary-thyroid (HPT) axis, upregulating hypothalamic thyrotropin-releasing hormone (TRH) expression and promoting thyroid T3 and T4 secretion, thereby enhancing appetite and energy expenditure [88,89,90,91,92,93]. Even within the normal physiological range, fluctuations in TH levels are closely associated with cold adaptation and the regulation of energy balance [94]. Dysregulation of TH function can occur under both malnutrition and overnutrition, with significant effects on energy metabolism and cardiovascular risk, highlighting the importance of long-term monitoring of thyroid homeostasis [95].

**Figure 3:**
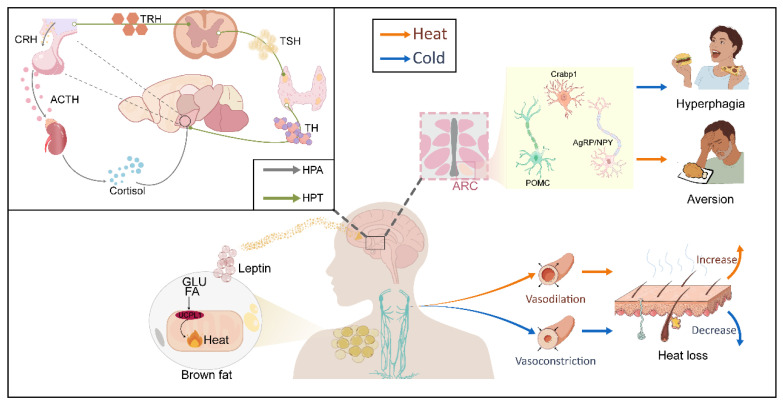
Temperature-driven plastic effector network coordinating energy intake, expenditure, and thermoregulation. This figure provides a systematic illustration of how temperature signals, after central integration, regulate whole-body metabolic homeostasis through a plastic effector network. The upper-left section depicts the activation of endocrine axes: temperature stimuli trigger the HPA axis to release CRH, ACTH, and cortisol, mobilizing energy; simultaneously, the HPT axis is activated, releasing TRH, TSH, and thyroid hormones, thereby enhancing basal metabolic rate and thermogenesis. The upper-right section focuses on central regulation of feeding behavior: POMC, AgRP/NPY, and Crabp1 neurons within the ARC integrate temperature and metabolic signals such as leptin, dynamically modulating appetite to produce either hyperphagic or anorectic responses. The lower section illustrates key peripheral effectors: BAT consumes energy and regulates metabolism directly through UCP1-mediated thermogenesis and the secretion of bioactive factors (e.g., leptin), while skin blood vessels precisely control heat dissipation via sympathetically mediated vasoconstriction and vasodilation to maintain thermal balance. Solid boxes/circles indicate selected regions, while dashed lines point to the corresponding magnified views. In the figure, red arrows indicate heat exposure or activation effects, whereas blue arrows represent cold exposure or inhibitory effects, visually depicting a dynamic network in which temperature signals drive energy intake, expenditure, and storage. This figure was created using Adobe Illustrator (version 2020) and Procreate (version 4.4.10).

In order to support the integrated control of body temperature and metabolic homeostasis, centrally integrated thermal signals send coordinated outputs to a flexible effector network that directs BAT thermogenesis, vasomotor tone, feeding behavior, and several endocrine axes. However, how different effectors are dynamically prioritized and coordinated under varying intensities and durations of thermal challenge remains unclear. Future studies should elucidate the key BAT-derived factors regulating distal metabolism; the temperature-specific responses and heterogeneity of different vascular beds; how temperature-dependent feeding interacts with overall energy status, modulated dynamically by seasonal and circadian rhythms; and the molecular switches that govern the transition of endocrine axes from acute adaptation to chronic dysregulation. Understanding the species-specific and individual variability of these mechanisms will provide a critical framework for translating basic discoveries into precise temperature-based interventions for the prevention and treatment of metabolic diseases (Figure 3).

## 5. Human Relevance and Metabolic Disease

The neuroendocrine and effector networks that couple temperature sensing to metabolic regulation constitute a core physiological system for maintaining energy homeostasis. In modern societies, however, widespread climate control and sedentary lifestyles markedly reduce exposure to periodic thermal stress, which may lead to insufficient engagement of this adaptive system and thereby represent an important environmental contributor to dysregulated energy balance and impaired glucose homeostasis [96].

### 5.1. Metabolic Syndrome and Environmental Temperature

MetS is a cluster of interrelated metabolic abnormalities, including obesity, insulin resistance or hyperglycemia, and dyslipidemia. Dysfunction of adipose tissue has emerged as a major driving factor and contributor to its pathogenesis. Collectively, these abnormalities increase the risk of cardiovascular disease and type 2 diabetes and are closely associated with dysregulation of energy balance and metabolic homeostasis [97].

Increasing evidence suggests that ambient temperature acts as a chronic environmental modulator of these processes. Observational studies provide compelling, albeit correlative, evidence for a link between ambient temperature and metabolic health. Epidemiological data indicate that environmental temperature is associated with the prevalence of obesity: populations exposed to higher ambient temperatures tend to exhibit higher body mass index [98]; controlled cold exposure and cold acclimation activate BAT, enhance energy expenditure, and improve insulin sensitivity [99]. In contrast, chronic residence in thermoneutral comfort conditions may promote “metabolic inertia,” thereby increasing the risk of obesity and MetS, whereas dynamic thermal exposure (moderate alternation between cold and warmth) may exert protective effects [100]. Overall, these findings suggest that environmental temperature modulates metabolic MetS by influencing adipose tissue function and systemic metabolic homeostasis.

### 5.2. Obesity and Energy Homeostasis

Obesity, as an adiposity-based chronic, relapsing disease, shares key characteristics with metabolic syndrome and is characterized by excessive, ectopic, and dysfunctional fat accumulation that harms other organs.

Accumulating evidence indicates that reduced BAT activity and diminished thermogenic responses contribute to decreased energy expenditure in obesity. In contrast, cold exposure serves as a key physiological stimulus that activates BAT and promotes energy dissipation via UCP1-dependent nonshivering thermogenesis [101,102,103]. This process is mediated by increased sympathetic outflow and represents an important mechanism linking environmental temperature to the regulation of body weight.

However, epidemiological observations remain complex. While some studies suggest that higher ambient temperatures are associated with increased body mass index [100], the interpretation of these data is complicated by heterogeneity in diagnostic criteria for abdominal obesity. International guidelines recommend using ethnicity-specific cut-off values for waist circumference (e.g., ≥85 cm for Chinese men [104] versus ≥102 cm for European men [97]) to define metabolic risk. This discrepancy in diagnostic thresholds creates a measurement bias, meaning that observed differences in obesity prevalence across regions may reflect methodological artifacts rather than purely biological responses to temperature.

Nevertheless, the underlying physiological link persists. Furthermore, reduced reliance on adaptive thermogenesis under warm conditions lowers energy expenditure and, if uncompensated by decreased food intake, may predispose individuals to fat accumulation [105]. Together, these findings highlight the critical role of thermoregulatory mechanisms in the development of metabolically unhealthy obesity, underscoring temperature as a key environmental modifier.

### 5.3. Diabetes and Glycemic Control

Beyond its established effects on energy balance and adiposity, environmental temperature critically governs glucose homeostasis. Experimental studies demonstrate that both acute and chronic moderate cold exposure can improve insulin sensitivity and glucose tolerance independently of changes in physical activity [106]. These metabolic benefits are largely mediated by activation of BAT and the browning of white adipose tissue [48,59,107]. Cold stimulation enhances glucose uptake and oxidation in BAT through largely insulin-independent mechanisms, contributing to improved systemic glycemic control.

Clinically, individuals with higher BAT activity exhibit superior glucose regulation [108] and more favorable lipid profiles [109,110]. In addition, cold-activated BAT secretes endocrine factors, including fibroblast growth factor 21 [111], interleukin-6 [112], and maresin-2 [113], which may further enhance glucose metabolism and attenuate obesity-associated inflammation. These findings suggest that thermogenic tissues function not only as energy dissipators but also as endocrine regulators of glucose homeostasis.

### 5.4. Complexity of Environmental Metabolic Regulation

While the metabolic benefits of cold exposure are increasingly documented, the impact of environmental heat on long-term metabolic outcomes remains poorly characterized. Most epidemiological studies have focused on cardiovascular strain [114,115,116], thermoregulatory responses [117], or heat-related mortality [118], with far fewer investigations systematically addressing how elevated ambient temperature influences resting energy expenditure, glucose homeostasis, insulin sensitivity, and adipose tissue function. Some population-based data suggest that higher environmental temperatures may be associated with an increased risk of metabolic disorders such as type 2 diabetes [119]. However, these observations are largely correlative and are susceptible to confounding by lifestyle, socioeconomic status, and behavioral factors, including physical activity and air-conditioning use. Consequently, it remains unclear whether heat exposure induces adaptive metabolic adjustments or instead promotes dysregulation of energy homeostasis.

In population studies, a central challenge lies in distinguishing causality from correlation. Most epidemiological evidence linking environmental temperature to metabolic disease remains inherently correlative, and outcomes are often confounded by long-term covariates such as geography, socioeconomic status, physical activity, diet, urbanization, and climate control practices [120,121,122,123,124]. For example, although populations exposed to higher ambient temperatures tend to exhibit higher body mass index [100], regional differences, including a higher prevalence of obesity in northern relative to southern China [125], suggest that these observations cannot be solely explained by environmental temperature.

By contrast, causal evidence is stronger in contexts where temperature exposure is experimentally manipulated or tightly controlled. Multiple cold-exposure intervention studies have shown that cold stimulation increases appetite, enhances thermogenesis [66,126], and improves insulin sensitivity [112], supporting a direct regulatory role of cold exposure on metabolic function. However, most mechanistic evidence comes from rodent models, which differ from humans in thermoregulatory strategies, BAT distribution, sympathetic innervation, and energy expenditure. These species differences highlight the need for validation of core mechanisms under controlled thermal conditions in humans.

Collectively, ambient temperature is unlikely to be a primary driver of metabolic dysregulation; rather, it appears to act as a modulatory factor shaping the sensitivity of metabolic pathways to nutritional and hormonal cues.

## 6. Conclusions and Future Perspectives

Ambient temperature has transcended its role as a mere background variable to emerge as a dynamic driver of metabolic plasticity. While the central nervous system integrates peripheral signals to coordinate autonomic and endocrine effectors, the modern “thermoneutrality” blunts these adaptive responses, predisposing individuals to metabolic dysregulation.

Despite technical advances, three questions remain unclear:

1. Do visceral organs possess intrinsic thermal sensors, or do they rely solely on propagating signals from the skin and core?
2. Does temperature regulate metabolism via direct cellular effects or predominantly through centralized neuroendocrine relays?
3. How do distinct physiological states (e.g., fasting, obesity) dynamically recalibrate the sensitivity of thermoregulatory circuits?

Future studies may bridge these gaps by integrating synchronous neural monitoring of skin and visceral afferents to decode signal propagation. Ultimately, elucidating these mechanisms will transition the field from correlative observation to precision intervention, establishing a novel strategy for optimizing energy homeostasis and treating metabolic disease.

## Data Availability

No new data were created or analyzed in this study. Data sharing is not applicable to this article.
